# Pectin-Coated Zeolitic Imidazolate Framework-8 Nanoparticles: A Dual-Responsive System for Controlled Carbendazim Delivery

**DOI:** 10.3390/ma18214961

**Published:** 2025-10-30

**Authors:** Yan Chen, Ragab Abouzeid, Qinglin Wu, Cornelis F. de Hoop, Jinqiu Qi

**Affiliations:** 1 School of Renewable Natural Resources, Louisiana State University Agricultural Center, Baton Rouge, LA 70803, USA; cy2259012925@gmail.com (Y.C.); rabouzeid@agcenter.lsu.edu (R.A.); 2 College of Forestry, Sichuan Agricultural University, Chengdu 611130, China

**Keywords:** carbendazim, nanofungicide, pectin, ZIF-8, controlled release, sweetpotato peels

## Abstract

The use of chemical fungicides in agriculture has led to the need for more efficient and sustainable solutions. Controlled-release nanomaterials offer a promising approach by improving fungicide delivery and reducing the need for frequent applications. This study investigates the synthesis of a dual-responsive nanofungicide through the loading of carbendazim (MBC) into zeolitic imidazolate framework-8 (ZIF-8), etching with tannic acid (TA) and the introduction of pectin (PT) to synthesize the MBC@ZTA-PT. The pectin, which was extracted from sweet potato peels, was applied as an eco-friendly, biodegradable additive that enhanced the stability and controlled-release properties of nanofungicide. Tannic acid etching significantly improved MBC loading efficiency. The cumulative release rates after 96 h under three different conditions were 33.12% at pH 7, 59.00% at pH 7 with the addition of pectinase, and 70.74% at pH 5 with the addition of pectinase, highlighting the strong responsiveness of the nanofungicide to pH and enzyme triggers. This dual-response system provided controlled release, thereby enhancing MBC utilization efficiency and minimizing the environmental hazards associated with fungicide applications. The findings suggest that MBC@ZTA-PT represents a promising, environmentally friendly strategy for sustainable plant disease management.

## 1. Introduction

Fungicides are an essential inputs in agricultural production, and they play a crucial role in disease control, yield enhancement, and crop quality. Among them, carbendazim (MBC), a highly effective broad-spectrum systemic benzimidazole fungicide, is widely applied owing to its high efficacy in preventing and managing fungal diseases such as Ascomycetes, Basidiomycetes, and Deuteromycetes [1]. In addition, MBC finds applications in the paint, textile, paper, and leather industries [2]. Its relatively low-cost and strong antifungal activity have made it one of the most widely used fungicides globally. However, traditional MBC formulations suffer from several limitations in field applications. They typically exhibit low utilization efficiency and are prone to environmental factors such as light, temperature fluctuations, microbial activity, and rainfall [3]. Such factors promote photolysis, microbial degradation, and leaching, which contribute to widespread loss of active ingredients and reduced field performance. Moreover, the high toxicity and non-specific mode of action of MBC create serious environmental and ecological concerns. Repeated use can lead to contamination of soil and aquatic environmental pollution, microbial community structure damage, and bioaccumulation in non-target organisms [1]. Residual MBC in agricultural produce also pose potential health risks to human beings [4]. Recent toxicological studies have confirmed that even low levels of MBC can lead to intestinal inflammation and barrier damage in non-target aquatic organisms [5]. Furthermore, MBC has been classified as having reproductive toxicity (Category 2) and exhibits potential carcinogenic properties; therefore, its application within the European Union is no longer permitted [6]. To address these issues, novel MBC delivery systems—such as hydrogels [7] or light-controlled release technologies [8,9]—have been proposed. Although these systems enhance the accuracy of targeting, reduce environmental losses, and lower ecological and health risks associated with traditional formulations, they generally have poor structural stability and are highly sensitive to environmental factors such as pH, temperature, and light.

Loading fungicides onto suitable nanocarriers has emerged as an effective strategy to achieve controlled and sustained release. Stimuli-responsive nanomaterials, such as those responsive to pH, temperature, enzymes, magnetic fields, and ultrasound, are particularly attractive for creating smart fungicide delivery systems. Among these, metal–organic frameworks (MOFs)—especially zeolitic imidazolate framework-8 (ZIF-8)—have attracted much attention in agriculture due to their high surface area, porosity, chemical and thermal stability, low cost, and biocompatibility [10,11]. ZIF-8, a subclass of MOFs composed of Zn^2+^ ions and 2-methylimidazole ligands, exhibits excellent pH responsiveness, degrading under acidic conditions due to protonation of the imidazole linkers [12]. This property makes it highly suitable for targeted fungicide delivery in acidic microenvironments, such as those formed by plant pathogens like Sclerotinia sclerotiorum, Botrytis cinerea, and Penicillium spp., which secrete organic acids such as oxalic, citric, and gluconic acids during infection [13,14]. Additionally, ZIF-8 decompose into bioavailable zinc ions, providing micronutritional benefits to crops while minimizing long-term environmental toxicity [10]. This dual function—controlled release and nutrient supplementation—positions ZIF-8 as a sustainable and eco-friendly nanocarrier for smart fungicide delivery.

Similarly, pectinase enzymes could be utilized as effective biological triggers for fungicide-controlled release systems. During infection, plant pathogens release various enzymes such as pectinases, cellulases, and proteases that break down the plant cell wall. This process helps the pathogens penetrate and spread within plant tissues [15,16,17]. Among these enzymes, pectinases play a particularly crucial role because they degrade pectin in the middle lamella, leading to the separation of plant cells and the initiation of cell death—a key step in the infection process of many pathogens. Based on this mechanism, pectin-modified nanocarriers have emerged as a promising strategy for enzyme-responsive delivery of fungicides [18,19,20,21]. Pectin (PT), a natural polysaccharide widely found in cell walls of plants and agriculture products, can form a protective coating around nanoparticles. This coating helps improve the retention, stability, and targeted release of fungicides in infected plant tissues where pectinase is present. When applied by spraying or coating on the plant surface, pectin-based formulations can provide long-lasting antimicrobial protection [22], thus reducing the overall amount of fungicide needed. Furthermore, pectin enhances the biodegradability of the nanocarrier system, minimizing environmental residues and ecological risks [23]. Since pectin is readily extracted from agricultural waste products such as citrus peels and sweet potato peels [24], its use also aligns with low-cost, sustainable, and eco-friendly material sourcing.

In this study, ZIF-8 was employed as a nanocarrier and etched with tannic acid (TA) to improve its loading capacity for MBC. Subsequently, pectin extracted from sweet potato peels was used to develop an environmentally friendly pectin-based coating, as previously reported for sustainable biopolymer applications [25,26]. The MBC@ZTA-PT nanoparticles were successfully synthesized and systematically characterized. Furthermore, the influence of pectinase activity and pH conditions on the release behavior of MBC from MBC@ZTA-PT nanoparticles was investigated. The results demonstrated that the MBC@ZTA-PT nanoparticles responded to both pectinase and acidic pH, enabling controlled and stimuli-triggered release of the fungicide. This smart delivery system provides prolonged and efficient protection against plant diseases, enhances the utilization efficiency of MBC, and significantly reduces environmental contamination.

## 2. Materials and Methods

### 2.1. Materials

Zinc nitrate hexahydrate (Zn(NO_3_)_2_⋅6H_2_O, 98% purity) and Carbendazim (97% purity) were procured from Strem Chemicals (Newburyport, MA, USA). 2-Methylimidazole (Hmim, 99% purity) was obtained from Acros Organics (Waltham, MA, USA). Methanol (ACS grade) was sourced from VWR Life Science (Solon, OH, USA). Tannic acid (TA, 95%) was purchased from Thermo Scientific (Waltham, MA, USA). EDC (1-(3-Dimethylaminopropyl)-3-ethylcarbodiimide Hydrochloride-98% purity) and NHS (N-Hydroxysuccinimide-98% purity) were provided by TCI (Tokyo Chemical Industry, Portland, OR, USA).

### 2.2. Extraction of Pectin from Sweetpotato Peels

Sweet potatoes (*Beauregard* variety) were collected from a local store in Baton Rouge, Louisiana, and thoroughly washed with deionized water to remove surface dirt and residual impurities. Approximately 500 g of fresh peels were soaked in 1.0 L of distilled water for 24 h at room temperature to promote softening and pre-extraction. Subsequently, the pH of the mixture was adjusted to 1.0 using hydrochloric acid, and the suspension was heated to 90 °C. This temperature was maintained for 2 h under constant stirring to facilitate efficient pectin extraction. After the extraction process, the mixture was subjected to rotary evaporation to remove insoluble solids and excess water to provide a concentrated pectin solution. Upon cooling to room temperature, ethanol was gradually added to the solution at a 1:2 (*v*/*v*) ratio to precipitate the pectin. The precipitate was collected by vacuum filtration, washed with ethanol, and then freeze-dried to obtain a purified pectin powder. The dried sample was ground into a fine powder using a mortar and pestle and stored in a desiccator for further use. The extraction yield of pectin was 10.87% on a dry-weight basis. The summarized SEM and FTIR analysis results are presented in Figure S1.

### 2.3. Synthesis and Characterization of MBC@ZTA-PT NPs

Initially, 1.46 g of Zn(NO_3_)_2_·6H_2_O and 3.24 g of Hmim were dissolved separately in 100 mL of methanol. The two solutions were then mixed and allowed to react under magnetic stirring at 700 rpm for 24 h at room temperature. The resulting solution was collected by centrifugation (10,000 rpm, 10 min), washed several times with methanol to remove residual impurities, and then dried at room temperature for 12 h to obtain ZIF-8 powder [27]. Next, 0.1 g of the obtained ZIF-8 powder was dispersed in 2 mL of deionized water via ultrasonication. Meanwhile, 0.25 g of TA was dissolved in 40 mL of deionized water. The ZIF-8 suspension was added to the TA solution and stirred for 15 min, followed by static aging for 1 h. The resulting mixture was then centrifuged, washed with deionized water, and air-dried for 12 h to yield light brown ZTA nanoparticles [27]. A total of 1 g of the ZTA nanoparticles was dispersed in 100 mL of methanolic carbendazim solution (1 g/L). The mixture was stirred magnetically for 24 h. Afterward, the solid was separated by centrifugation, washed, and dried to obtain MBC@ZTA. A total of 1 g of MBC@ZTA and 0.3 g of NHS were dispersed in 100 mL of methanol. Simultaneously, 0.2 g of pectin and 0.3 g of EDC were dissolved in 50 mL of deionized water. The pectin solution was poured into the MBC@ZTA solution and stirred magnetically for 24 h. This EDC/NHS coupling chemistry is widely used to modify the surface of nanomaterials with biological molecules [28]. In this process, EDC serves as a carbodiimide coupling agent that activates the carboxyl groups of pectin, while NHS stabilizes the intermediate and promotes its reaction with amino groups on the ZTA surface to form amide bonds, resulting in a stable pectin coating. Finally, the resulting mixture was centrifuged at 10,000 rpm for 10 min and the precipitate was collected, washed twice with methanol, and dried in an oven at 50 °C to prepare MBC@ZTA-PT nanoparticles.

The morphology of the prepared samples (ZIF-8, ZTA, MBC@ZTA, and MBC@ZTA-PT) was examined using a Field Emission Scanning Electron Microscope (Quanta 3D DualBeam FEG FIB-SEM, FEI Company, Hillsboro, OR, USA). Prior to SEM analysis, the samples were platinum-coated using a sputter coater. Fourier Transform Infrared (FTIR) spectroscopy was performed with an Agilent Cary 630 FTIR spectrometer (Agilent Technologies, Santa Clara, CA, USA). Spectra were collected in the wavenumber range of 4000–400 cm^−1^ with a resolution of 4 cm^−1^. X-ray diffraction (XRD) analysis of the samples was carried out using an Empyrean diffractometer (Malvern Panalytical Ltd., Great Malvern, UK) with Cu Kα radiation (λ = 1.5406 Å). Measurements were conducted over a 2θ range of 5–80° with a step size of 0.04°. The zeta potential of the suspensions was determined using a Nano ZS Ultra Analyzer (Malvern Panalytical Inc., Westborough, MA, USA). Five measurements were taken for each sample, and the average values were reported. Thermal stability of the prepared samples was assessed by thermogravimetric analysis (TGA) using a Q50 analyzer (TA Instruments Inc., New Castle, DE, USA). The analyses were performed under a nitrogen atmosphere with a temperature range of 30–600 °C, a heating rate of 10 °C/min, and a sample weight of less than 5 mg.

### 2.4. In Vitro Release of MBC@ZTA-PT

The cumulative release profiles of MBC from MBC@ZTA-PT were investigated under different pH conditions (pH 7 and pH 5) and in the presence or absence of pectinase (1 mg/mL) to assess their dual-stimuli-responsive release behavior. A total of 100 mg of MBC@ZTA-PT NPs were dispersed in 500 mL of methanol–water solution (*v*/*v* = 50:50) under continuous magnetic stirring at 300 rpm. At predetermined time intervals (1, 2, 3, 4, 5, 12, 24, 48, 72, and 96 h), 2 mL of the supernatant was collected, and an equal volume of release medium was added. The collected supernatant was filtered through a 0.22 μm membrane and analyzed for MBC concentration using a UV-Vis spectrophotometer. A full-wavelength scan was conducted in the wavelength range of 200–400 nm to determine the optimal testing wavelength for MBC. The pH values of 7 and 5 were adjusted manually using dilute HCl and NaOH solutions. All experiments were performed in triplicate to ensure reproducibility.

In the UV-Vis spectrum of MBC@ZTA-PT, a distinct absorption peak at 290 nm was observed, confirming the successful loading of MBC onto the composite material. This absorption peak corresponds to the typical UV absorption characteristics of MBC, mainly attributed to the π-π* electronic transition of its aromatic structure. A calibration curve (y = 0.0253x + 0.177, R^2^ = 0.999) was established by measuring the absorbance of MBC standard solutions with gradient concentrations at 290 nm. The absorbance of the composite was then measured under the same conditions, and the background absorption from ZTA was subtracted. Based on the corrected absorbance values, the actual MBC content in the solution was accurately determined.

The fungicide loading capacity (FLC) was determined by dispersing 100 mg of the sample in a methanol–water solution, followed by the addition of pectinase and pH adjustment. The mixture was ultrasonicated for 40 min to ensure complete release of MBC. Then the supernatant was filtered through a 0.22 μm membrane, and the MBC concentration in the filtrate was measured using a UV-Vis spectrophotometer. The FLC was calculated based on a standard curve. The FLC was calculated using the following formula [27]:

(1) $$\mathrm{FLC}\;(\%)\;=\;\frac{m}{M}\times \;100\%$$

where m represents the mass of MBC loaded in the sample and M is the total mass of the drug carrier (100 mg).

Drug release kinetics were evaluated using zero-order, first-order, Korsmeyer–Peppas, Higuchi, and logistic growth models. The model with the regression coefficient R^2^ closest to 1 was identified as the best-fitting model. The cumulative release percentage of MBC was determined using the following equation [27]:

(2) $$\mathrm{Cumulative}\;\mathrm{release}\;(\%)\;=\;\frac{C_{t}\;\times \;V_{0}\;+\;\sum_{0}^{t}C_{t}\;\times \;V}{M}\times \;100$$

where Ct represents the concentration in the sample at time t, V_0_ is the total volume of the release medium (500 mL), V is the volume of the sample collected at each time point (2 mL), and M is the total amount of MBC loaded in 100 mg of the sample.

### 2.5. Release Kinetics Investigation

The release kinetics of MBC from the MBC@ZTA-PT system were evaluated using five mathematical models, including the zero-order, first-order, Korsmeyer–Peppas, Higuchi, and logistic growth models [27,29]. The equations for each model are as follows:

Ct = kt + C0(3)

ln(Ct) = −kt + ln(C0)(4)

lCt/Cmax = k·tn(5)

lQt = k√t + C(6)

(7) $$l\mathrm{Ct}=\frac{\mathrm{Cmax}}{1+{\;e}^{-k\;(t-t0)}}$$

where Ct represents the concentration of the released drug at time t, Cmax is the maximum concentration, k and C are constants, n is the release exponent that characterizes the release mechanism, Qt is the cumulative amount of drug released at time t, and t0 is the time at which half of the maximum concentration is reached.

### 2.6. Statistical Analysis

The data were analyzed using IBM SPSS Statistics, Version 26.0 (IBM Corp., Armonk, NY, USA; last updated 2019), and statistical significance was determined via Duncan’s test (*p* < 0.05). The reported values represent the means of three replicates.

## 3. Results

### 3.1. Characteristics of Nanoparticles

**Figure 1:**
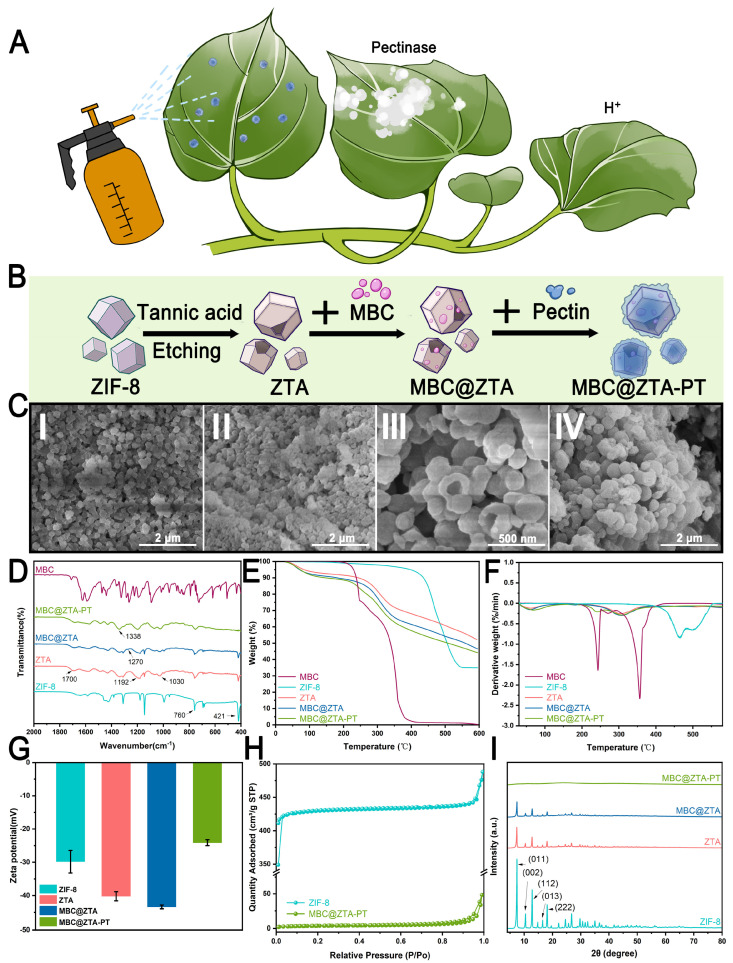
Synthetics and characterization of nanoparticles. (**A**) Application schematic. (**B**) Schematic mechanism of the synthesis process for MBC@ZTA-PT nanoparticles. (**C**) SEM images of ZIF-8 (I), ZTA (II), MBC@ZTA (III), MBC@ZTA-PT (IV). (**D**) FTIR, (**E**) TGA, (**F**) DTG, (**G**) zeta potential, (**H**) N2 adsorption–desorption isotherms, and (**I**) XRD characterization of MBC, ZIF-8, ZTA, MBC@ZTA, and MBC@ZTA-PT.

The success of pectin extraction from waste sweet potato peels was confirmed by comparing the infrared spectra of the extracted pectin with those of commercial pectin powder. The characteristic peaks were consistent, validating the successful extraction of pectin, with a yield of 10.87%. Figure 1A illustrates a simulated application scenario where nanoparticles are sprayed onto plant surfaces. Given that fungi secrete pectinase during infection and simultaneously cause a localized pH decrease at the infection site, the samples are engineered to respond to the dual stimuli of pectinase activity and low pH, enabling the controlled release of MBC. This strategic design of targeted release ensures precise delivery of the MBC to the areas affected by the infection while also minimizing environmental impact. Figure 1B schematically illustrates the fabrication process of MBC@ZTA-PT nanoparticles. The morphological evolution of the samples at each stage of synthesis was systematically characterized by SEM, as shown in Figure 1C. The loading capacity of MBC varied with different ratios of TA etching. Specifically, the loading amounts were 25.45% for a ZIF-8:TA ratio of 1:1, 45.65% for 1:2.5, and 46.76% for 1:4. Although a higher TA ratio slightly increased the loading capacity, excessive etching has been reported to damage the structural integrity of ZIF-8, as demonstrated in Yang’s study [30]. Therefore, the optimal ZIF-8:TA ratio of 1:2.5 was selected to achieve a balance between high loading efficiency and structural stability. After etching, the surface of ZIF-8 became markedly rougher and uneven, with numerous pores and cracks exposed. This structural alteration significantly increased the specific surface area of ZIF-8, enhancing both its fungicide loading capacity and surface reactivity (Figure 1C(II)). Notably, MBC loading failed to affect morphological characteristics (Figure 1C(III)). However, the coating of pectin transformed the particle surfaces into larger, flower-like or spherical structures, which showed the successful creation of the composite material (Figure 1C(IV)). Figure 1D presents the FTIR spectra of the samples, elucidating the chemical structural characteristics and interactions among the components. The ZIF-8 spectrum shows characteristic peaks at 760 cm^−1^ and 421 cm^−1^. These peaks correspond to Zn-N stretching vibrations, which confirmed the successful coordination of zinc ions with imidazole ligands within the ZIF-8 framework [31]. After TA etching, the FTIR spectrum of ZTA exhibited a new absorption peak at 1700 cm^−1^, corresponding to the C=O stretching vibration of the phenolic carboxyl groups in TA [32]. Additionally, characteristic bands at 1192 cm^−1^ and 1030 cm^−1^ are attributed to C–O stretching vibrations within the aromatic polyphenol structure of TA, confirming its successful integration onto the ZIF-8 surface. In the MBC@ZTA spectrum, the peak at 1270 cm^−1^, corresponding to the C-O bond vibration of the benzimidazole ring in MBC, and a general decrease in peak intensity suggest successful MBC loading. There are also new peaks related to C-N and C=O vibrations, aligning with MBC’s molecular structure, which verify its integration into the composite [33]. In the MBC@ZTA-PT spectrum, the increased peak strengths at 1030 cm^−1^ and 1338 cm^−1^, corresponding to the C-O and C-OH stretching vibrations, respectively [34], confirm the successful addition of pectin, highlighting its effective coating on the composite material. Figure 1E,F present the TGA of the synthesized nanoparticles, including MBC, ZIF-8, ZTA, MBC@ZTA, and MBC@ZTA-PT, to evaluate their thermal stability. The residual mass percentages at 600 °C were determined to be 0.30%, 34.94%, 52.18%, 46.50%, and 40.19%, respectively. The initial weight loss observed below 150 °C was mainly due to the evaporation of physically adsorbed water. Pure MBC exhibited rapid weight loss beginning at 180 °C, with major decomposition peaks at around 240 °C and 350 °C, indicating its relatively poor thermal stability [33]. In contrast, ZIF-8 demonstrated better thermal stability, retaining most of its mass up to 400 °C, consistent with previous reports of its stability beyond 350 °C. The modification of ZIF-8 through TA etching introduced hydroxyl (-OH) and carboxyl (-COOH) functional groups [35], promoting earlier decomposition and slightly increasing the thermal stability [36]. The significant weight loss observed between 200 and 400 °C in MBC@ZTA is consistent with the thermal degradation range of free MBC, confirming its successful loading into the framework. Pectin addition into MBC@ZTA-PT led to an additional minor weight loss between 200 and 250 °C because of the degradation of the polysaccharide backbone [37]. This pattern also supports the successful coating of the composite with pectin and its partial thermal contribution. Figure 1G displays the evolution of zeta potential throughout the stepwise synthesis of the nanocomposites. Initially, the zeta potential of pristine ZIF-8 was approximately −29.82 mV, which is consistent with previously reported values for unmodified ZIF-8, consistent with surface deprotonation of imidazole ligands [38]. Following etching with TA, the zeta potential significantly decreased to −43.41 mV. This enhanced increase in surface negativity can be attributed to the introduction of additional carboxyl and phenolic hydroxyl groups from TA, which enhance the surface charge density through increased ionization at a neutral pH [39]. Subsequent incorporation of PT caused a significant reduction in the absolute value of the zeta potential to −24.10 mV. This trend likely resulted from the neutralization and shielding of charged residues by polysaccharide chains [40]. Figure 1H presents the N_2_ adsorption–desorption isotherms and the corresponding pore size distributions of the samples. ZIF-8 exhibits a typical Type I isotherm, indicative of a predominantly microporous structure. The surface area, adsorption average pore diameter, and average particle size for ZIF-8 were 1301.2476 m^2^/g, 2.1041 nm, and 4.6110 nm, respectively. In contrast, MBC@ZTA-PT exhibited lower adsorption capacities, especially in the low-pressure region, with their isotherms demonstrating characteristics of a typical Type IV pattern, suggesting a predominantly mesoporous structure. Upon etching with TA and subsequent incorporation of MBC and PT, significant alterations were observed in the porous characteristics of MBC@ZTA-PT. The adsorption average pore diameter and average particle size notably increased to 6.7671 nm and 483.4183 nm, respectively, while the surface area decreased substantially to 12.4116 m^2^/g. Figure 1I presents the XRD patterns of the synthesized samples to assess their crystallinity and structural evolution during the modification process. Pristine ZIF-8 exhibits sharp diffraction peaks at 2θ values of 7.4°, 10.4°, 12.7°, 16.5°, and 18.0°, which correspond to the (011), (002), (112), (013), and (222) planes, respectively—typical of the sodalite-type crystalline framework of ZIF-8 [31,41]. Following TA etching, the XRD pattern of ZTA shows no new diffraction peaks, consistent with the amorphous nature of TA. Although the ZIF-8 structure remains identifiable, a noticeable decrease in peak intensity was observed in both ZTA and MBC@ZTA, indicating partial structural disruption and an increased proportion of amorphous components on the surface. Upon the application of a pectin coating, the XRD pattern of MBC@ZTA no longer displayed distinct ZIF-8 characteristic peaks but instead showed broad diffraction peaks. This broadening can be attributed to pectin, a highly amorphous natural polymer, which likely formed a thick coating on the surface. This thick amorphous layer may have obscured the diffraction signals from the underlying ZIF-8 crystalline structure, preventing their effective detection through the pectin layer.

**Figure 2:**
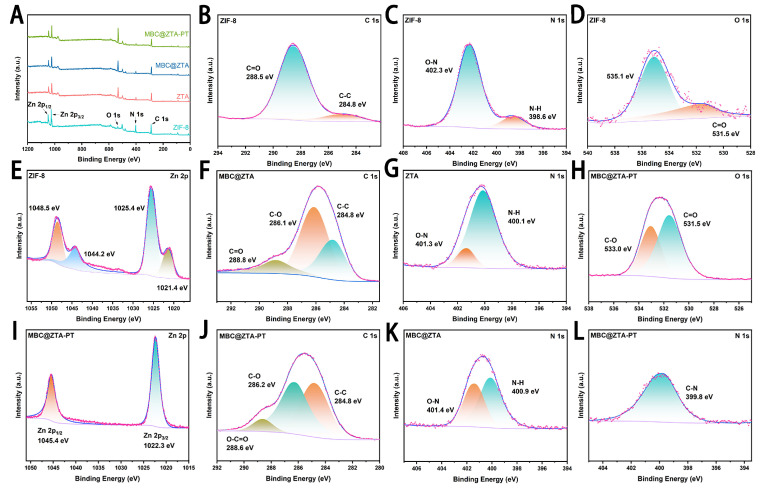
(**A**) Analyses of ZIF-8, ZTA, MBC@ZTA and MBC@ZTA-PT. (**B**) C 1s (**C**) N 1s (**D**) O 1s and (**E**) Zn 2p spectra of the ZIF-8. (**F**) C 1s spectra of the MBC@ZTA, and (**G**) N 1s spectra of the ZTA. (**H**) O 1s, (**I**) Zn 2p, and (**J**) C 1s spectra of the MBC@ZTA-PT. (**K**) N 1s spectra of the MBC@ZTA. (**L**) N 1s spectra of the MBC@ZTA-PT.

The elemental composition and surface electronic environment of the MBC@ZTA-PT nanoparticles were analyzed using XPS, and the results are shown in Figure 2. The survey spectrum displayed characteristic core-level binding energies at 284.8 eV (C 1s), 398.7 eV (N 1s), 531.2 eV (O 1s), and 1021.1 eV (Zn 2p), confirming the presence of the main elements in the composite. High-resolution XPS spectra were further employed to characterize the interactions among ZIF-8, TA, and pectin, focusing on the Zn 2p, C 1s, O 1s, and N 1s binding energies (Figure 2B–L). To compensate for charging effects, all binding energy measurements were calibrated using the C 1s peak at 284.80 eV as a reference. The XPS binding energy represents the energy required to remove core electrons from atomic orbitals, which differs fundamentally from chemical bond dissociation energies. Therefore, any observed shifts in XPS peaks arise from alterations in the electronic environment or oxidation state of atoms, rather than from changes in chemical bond strength [42]. The Zn 2p XPS spectrum of ZIF-8 exhibits broad peaks at 1025.4 eV (Zn 2p_3_/_2_) and 1048.5 eV (Zn 2p_1_/_2_), along with additional peaks, suggesting the coexistence of Zn in both Zn-N and Zn-O environments. In contrast, the Zn 2p peaks in MBC@ZTA-PT become significantly narrower, with a noticeable blue shift to 1022.3 eV and 1045.4 eV. This shift indicates that Zn experiences stronger electron attraction, leading to a higher oxidation state and a more homogeneous chemical environment. These results agree well with those reported for a prochloraz (Pro) and pH-jump reagent-loaded zeolitic imidazolate framework-8 (PD@ZIF-8) by Liang et al., where a similar peak shift in Zn 2p peaks was attributed to the substitution of N-ligands with O-donor groups from loaded fungicides. Such shifts indicate electron withdrawal from Zn centers, producing a more polarized Zn–O–C environment [9]. The valence state distribution of Zn was further analyzed by measuring the spin–orbit energy separation (ΔE = 23.1 eV) between the Zn 2p_3_/_2_ and Zn 2p_1_/_2_ peaks. This value is consistent with that reported for Zn^2+^ species in Zn-based metal–organic frameworks, such as ZIF-8 and ZnO, and significantly different from that of metallic Zn [43,44]. The C 1s XPS spectrum further reveals the evolution of surface functional groups. ZIF-8 primarily consists of C-C (284.8 eV) and C=O (288.5 eV) bonds, indicating a low content of oxygen-containing functional groups. After etching and MBC loading, the appearance of a C-O bond (286.1 eV) suggests surface oxidation introducing ether or hydroxyl groups. With the subsequent addition of pectin, MBC@ZTA-PT undergoes further oxidation, as evidenced by the emergence of an O-C=O peak (288.6 eV), indicating the formation of carboxyl or ester groups.

### 3.2. Pectinase and pH-Responsive Release Performance

**Figure 3:**
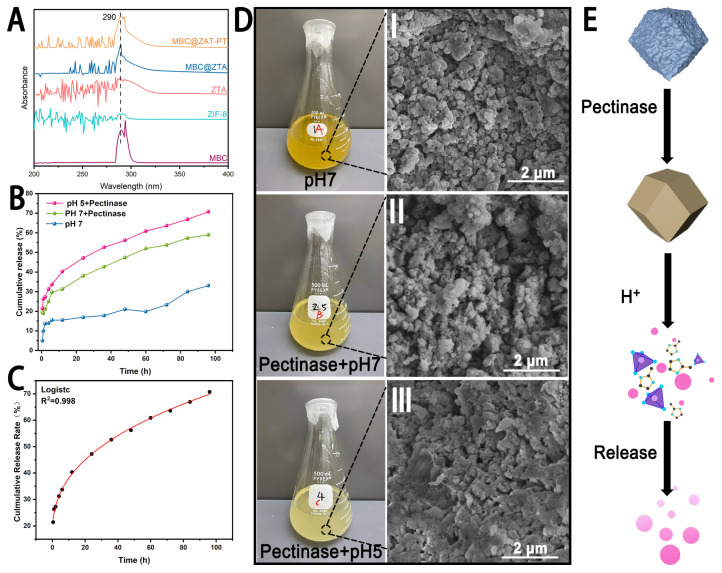
(**A**) Characteristic Absorption Peaks of MBC (under the same release conditions). (**B**) Release behavior of MBC@ZTA-PT. (**C**) Logistic growth model of cumulative release kinetics at pH 5 with pectinase. (**D**) Solution color and SEM analysis of MBC@ZTA-PT after release under different conditions. (**E**) Schematic of dual-response drug release mechanism.

The cumulative release profiles of MBC@ZTA-PT under different environmental conditions are illustrated in Figure 3B. After 96 h, the cumulative release rates under the pH 7, pH 7 with pectinase, and pH 5 with pectinase conditions were 33.12%, 59.00%, and 70.75%, respectively, indicating that MBC@ZTA-PT NPs exhibited a significantly higher release rate in an acidic environment supplemented with pectinase. This phenomenon can be attributed to the enzymatic degradation of pectin, in which pectinase hydrolyzes the polysaccharide chains within pectin, thereby disrupting its structure and reducing its physical constraint on drug molecules, ultimately accelerating MBC release. Moreover, the pH-responsive characteristics of MBC@ZTA-PT NPs are closely related to their structural stability. During the degradation of pectin, the exposed ZTA framework gradually disintegrates due to the protonation of imidazole ligands under acidic conditions, leading to the breakdown of Zn^2+^-imidazole coordination and subsequent structural collapse of ZTA [30], thereby facilitating the release of MBC from MBC@ZTA-PT. This suggests that MBC@ZTA-PT NPs can be activated in response to the fungal microenvironment, enabling targeted and sustained MBC release, thereby enhancing therapeutic efficacy. This dual-responsive drug release system can effectively prolong drug release, reduce environmental loss, and improve fungicidal efficiency. To investigate the release mechanism of MBC@ZTA-PT Nps, cumulative release data were fitted using five models: zero-order, first-order, Korsmeyer–Peppas, Higuchi, and the logistic growth models. The results, shown in Table 1, indicate that the logistic growth model provided the best fit, followed by the Korsmeyer–Peppas model. In contrast, the zero-order, first-order kinetics, and Higuchi models yielded slightly lower R^2^ values, indicating a less satisfactory fit. Based on detailed analysis of the data, the logistic growth model was determined to be the most suitable for describing the drug release process. As illustrated in Figure 3C, this model not only showed a high degree of fit but also captured the S-shaped growth characteristics of the release profile. Additionally, external factors such as pectinase and pH significantly influenced the drug release. The incorporation of pectinase and the reduction in pH values notably increased both the release amount and the release rate of the drug. According to the Korsmeyer–Peppas model, when the release exponent (n) is ≤0.5, the drug release follows a Fickian diffusion mechanism [45], indicating that MBC@ZTA-PT Nps primarily releases through simple diffusion. Under different environmental conditions, interesting changes in the color of the MBC@ZTA-PT suspension were observed: after 96 h of release, the suspension at pH 7 appeared to be the darkest brown; upon the addition of pectinase, the suspension color at pH 7 turned medium yellow; and in a pH 5 environment with pectinase, the color shifted to light yellow (Figure 3D). Correspondingly, the morphological structure of MBC@ZTA-PT Nps also exhibited distinct changes under different release conditions. Under neutral conditions, the MBC@ZTA-PT Nps maintained their structural integrity. However, in the presence of pectinase, slight dissolution was observed on the surface of the particles. In contrast, under pH 5 with pectinase, significant structural degradation occurred, with partial dissolution and collapse of the particles. Figure 3E illustrates a schematic of the dual-response drug release mechanism: under the action of pectinase, the outer pectin layer gradually dissolves, exposing the ZTA structure, which further decomposes in the acidic environment, thereby releasing more MBC.

## 4. Conclusions

In this study, a novel MBC-loaded nanomaterial (MBC@ZTA-PT) was designed with ZIF-8 and pectin. The pectin, extracted from sweet potato peels, with a yield of 10.87%, acted as an eco-friendly and biodegradable component. It improved the stability and controlled-release performance of the nanofungicide. The introduction of TA etching significantly improved the surface roughness and porosity of ZIF-8, resulting in a higher MBC loading efficiency. The cumulative release rates after 96 h under different conditions were 33.12% at pH 7, 59.00% at pH 7 with the addition of pectinase, and 70.74% at pH 5 with the present of pectinase, demonstrating its excellent dual responsiveness of MBC@ZTA-PT to both enzymatic and acidic environments. These findings highlight that MBC@ZTA-PT provides an effective and sustainable delivery platform for precise, controlled fungicide release. The combination of ZIF-8′s pH responsiveness with pectin’s enzyme sensitivity ensures efficient utilization of MBC while reducing environmental residues. In general, the design strategy developed in this study can be extended to other fungicide molecules. It provides a versatile and eco-friendly platform for developing stimuli-responsive nanocarriers. Such systems have great potential for precise fungicide delivery and reducing chemical use. They also help promote sustainable agricultural, and support the development of green and smart plant protection, technologies.

## Figures and Tables

**Table 1:** Comparison of five kinetic models for MBC@ZTA-PT release at pH 5 with pectinase.

Model		pH = 7	Pectinase + pH = 7	Pectinase + pH = 5
Zero-order	k	0.21	0.42	0.43
	C0	11.15	23.76	28.25
	R^2^	0.86	0.94	0.95
First-order	k	−0.01	−0.01	−0.01
	ln(C0)	2.44	3.18	3.35
	R^2^	0.86	0.86	0.88
Korsmeyer–Peppas	k	8.68	18.11	22.99
	n	0.25	0.25	0.24
	R^2^	0.83	0.98	0.99
Higuchi	k	2.11	2.86	2.98
	C	7.66	19.42	24.52
	R^2^	0.85	0.92	0.93
Logistic Growth Model	t0	60.15	47.85	54.52
	k	0.04	0.03	0.03
	R^2^	0.95	0.99	0.99

## Data Availability

The original contributions presented in this study are included in the article/Supplementary Material. Further inquiries can be directed to the corresponding authors.

## Supplementary Materials

The following supporting information can be downloaded at https://www.mdpi.com/article/10.3390/ma18214961/s1. Figure S1. Summary of the SEM and FTIR characterization results of the self-extracted pectin.

## Abbreviations

The following abbreviations are used in this manuscript:

MBC	carbendazim
ZIF-8	zeolitic imidazolate framework-8
TA	tannic acid
PT	pectin
MOFs	metal–organic frameworks
