# Supplementary figures and images for: Pectin-Coated Zeolitic Imidazolate Framework-8 Nanoparticles: A Dual-Responsive System for Controlled Carbendazim Delivery
Source: Materials (Basel). 2025 Oct 30;18(21):4961. doi: 10.3390/ma18214961 (PMC12608521; doi:10.3390/ma18214961)

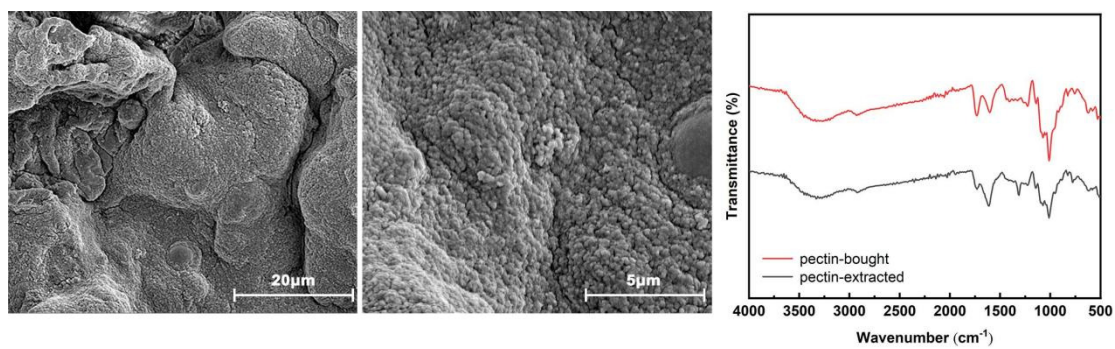

**Figure S1.** Summary of the SEM and FTIR characterization results of the self-extracted pectin.

Supplement: Supplementary file 1 [file materials-18-04961-s001.zip › materials-3935816-supplementary.pdf]
